# Optimization of OSEM parameters in myocardial perfusion imaging reconstruction as a function of body mass index: a clinical approach*

**DOI:** 10.1590/0100-3984.2014.0084

**Published:** 2015

**Authors:** Pietro Paolo de Barros, Luis F. Metello, Tatiane Sabriela Cagol Camozzato, Domingos Manuel da Silva Vieira

**Affiliations:** 1 Radiology Technologist at Clínica Imagem Centro de Diagnóstico, Graduated at Instituto Federal de Educação, Ciência e Tecnologia de Santa Catarina (IFSC), Florianópolis, SC, Brazil.; 2 Master, Nuclear Medicine Technologist, Professor at Escola Superior de Tecnologia em Saúde do Porto – Instituto Politécnico do Porto (ESTSP-IPP), Porto, Portugal.; 3 PhD, Professor at Instituto Federal de Educação, Ciência e Tecnologia de Santa Catarina (IFSC), Florianópolis, SC, Brazil.; 4 Fellow Master degree of Medical Information Technology, School of Medicine – Universidade do Porto (FMUP), Professor at Escola Superior de Tecnologia da Saúde do Porto – Instituto Politécnico do Porto (ESTSP-IPP), Porto, Portugal.

**Keywords:** Nuclear cardiology, Myocardial perfusion imaging, Iterative methods, Image reconstruction, Nuclear medicine

## Abstract

**Objective:**

The present study is aimed at contributing to identify the most appropriate OSEM parameters to generate myocardial perfusion imaging reconstructions with the best diagnostic quality, correlating them with patients’ body mass index.

**Materials and Methods:**

The present study included 28 adult patients submitted to myocardial perfusion imaging in a public hospital. The OSEM method was utilized in the images reconstruction with six different combinations of iterations and subsets numbers. The images were analyzed by nuclear cardiology specialists taking their diagnostic value into consideration and indicating the most appropriate images in terms of diagnostic quality.

**Results:**

An overall scoring analysis demonstrated that the combination of four iterations and four subsets has generated the most appropriate images in terms of diagnostic quality for all the classes of body mass index; however, the role played by the combination of six iterations and four subsets is highlighted in relation to the higher body mass index classes.

**Conclusion:**

The use of optimized parameters seems to play a relevant role in the generation of images with better diagnostic quality, ensuring the diagnosis and consequential appropriate and effective treatment for the patient.

## Abstract (pt)

**Objetivo:**

O presente trabalho visa contribuir para identificar quais os parâmetros OSEM que geram as reconstruções na cintilografia de perfusão do miocárdio com o maior valor clínico de diagnóstico, relacionando-os com as classificações de índice de massa corporal.

**Materiais e Métodos:**

Foram selecionados 28 pacientes adultos que realizaram cintilografia de perfusão do miocárdio em um hospital público. Cada paciente teve seu exame processado com o método OSEM em seis combinações diferentes de número de iterações e subsets. As imagens foram analisadas por especialistas em cardiologia nuclear, que consideraram o valor clínico de diagnóstico delas e indicaram quais as imagens que mais se adequavam à qualidade diagnóstica.

**Resultados:**

Em análise global da pontuação conforme as avaliações médicas, o arranjo de quatro iterações e quatro subsets gerou as imagens com melhor qualidade diagnóstica em todas as classes de índice de massa corporal, porém o arranjo com seis iterações e quatro subsets se destacou nas classes de maiores índices de massa corporal.

**Conclusão:**

A utilização de parâmetros otimizados parece ter papel importante em proporcionar reconstruções de melhor qualidade diagnóstica, garantindo-se assim que o paciente tenha o seu diagnóstico e consequente terapêutica encaminhados de forma mais adequada e eficaz.

## INTRODUCTION

According to statistics from the World Health Organization (WHO), the number of deaths caused by cardiovascular problems in developed countries is much higher than the number of deaths caused by diseases such as malignant neoplasms and respiratory disorders. Approximately 12 million people in the world die every year from heart attack or infarction, either directly or indirectly caused by related complications. Currently, it is estimated that the number of deaths caused by cardiovascular diseases has increased to 17.5 million per year. Such deaths occur in all social classes and are commonly associated with the male gender and age above 40. In truth, both men and women may be affected by heart attacks, and women face higher risks after menopause. Cardiovascular diseases were responsible for 30% of the 58 million deaths occurred in 2008. Countries with low and medium income are more affected, accounting for 80% of such deaths^(1-5)^.

In nuclear medicine, studies of the cardiovascular system are utilized to detect and characterize conditions such as coronary artery disease, for example. According to Strauss et al.^(6)^, although nuclear procedures may be utilized in order to characterize congenital diseases, such studies are, generally, performed in combination with echocardiography and magnetic resonance imaging.

Myocardial perfusion imaging (MPI) is the main procedure in nuclear cardiology, accounting for more than 75% of cardiac studies in many centers. It presents the advantage of being a robust procedure, and is reproducible to a certain extent.

As the patient is injected with radioactive material, one should consider that not all photons leave the human body or remain in their initial trajectory due to interactions with the patient's tissues. Such a factor is more significant in patients with higher body mass indexes (BMI), as in such patients there is a greater amount of tissues and radiation attenuating structures, which may generate artifacts interfering in the quality of the resulting images^(7,8)^.

Myocardial perfusion images provide relevant functional data for appropriate characterization of the diseases and their treatment, and its final appearance results from the interaction of various processes. Despite the availability of several methods for correction of motion, attenuation and scattering, the related programs easily show their limitations. For that reason, the processing is highly dependent on the consistency of the raw data obtained during the images acquisition. Based on data acquired by the gamma chamber detectors, the purpose of tomographic images reconstruction is to generate an image depicting a true sectional view of an object^(7,9,10)^.

The theory on mathematical image reconstruction dates from 1917, when the Austrian mathematician Johann Radon published his study demonstrating how the original function could be determined from a set of projections. Since then, many tomographic image reconstruction algorithms (single photon emission computed tomography - SPECT) have been developed and proposed for images processing in nuclear medicine^(11)^. Among such algorithms, the most common ones are the analytical images reconstruction algorithms and the iterative methods^(8,12)^.

In nuclear medicine, analytical methods constitute the simplest technique for tomographic reconstruction. One of such methods is filtered back projection, which basically consists of the filtering of projections in the frequency domain by utilizing a ramp filter and, after performing the inverse transformation, retroproject the data to generate the final image^(13)^.

According to Lalush et al.^(14)^, the iterative methods were formally proposed in 1977 as a solution for statistical problems involving incomplete data. The basic functioning principle of iterative algorithms is, by means of successive estimates, minimizing the difference between the projections measured and estimated by the algorithm. The existing iterative algorithms differ particularly in the way the measured and estimated projections are compared, besides the types of corrections applied to the current estimate. Such a process may be initiated by creating an initial estimation with the value 0 or 1, depending on whether the correction is performed by means of addition or multiplication, respectively^(8,15)^.

Based on simpler iterative methods, other methods were developed, considering a given criterion optimized to generate the best estimate for the problem solution. The maximum-likelihood (ML) criterion is a standard utilized in statistics, proposed by R. A. Fisher in 1921, and provides a prescription to decide, among all possible images, which is the best estimate of the true object. Among the developed methods the maximum-likelihood expectation maximization (MLEM) method is highlighted.

The MLEM algorithm aims at finding a general solution as the best estimate of the mean number of radioactive disintegrations on the image, which can produce a sinogram with the greatest likelihood.

Such a solution is generated by means of statistical models, which allow for the prediction of the probability of a number of detected counts for a given mean number of disintegrations. Each iteration is divided into two parts called expectation (E) and maximization (M). In the E part of the iteration, the formula that expresses the likelihood of any reconstructed image from measured data is formed. In the M part, the image with the greatest likelihood with the measured data is obtained.

As the process of the MLEM algorithm convergence is quite slow, its utilization in clinical context would not be appropriate. Thus, in 1994, Hudson and Larkin proposed the ordered-subset expectation maximization (OSEM) algorithm, a variation of the MLEM method, whose objective is accelerating the image reconstruction process by using such an algorithm.

The basic principle of OSEM consists of dividing the total set of projections into smaller subsets. Each subset has the same number of projections. In addition, the number of subsets that can be created is a multiple of the total number of projections. For example, if during a scan 64 projections are acquired from a patient (representing 64 different angles), they can be divided into subsets, each one containing 8 images. The 64 projections may also be divided into 16 subsets, each one containing 4 images. After distribution of the projections in subsets, the MLEM algorithm is applied to all sets of projections, each one corresponding to a fraction of the iteration. When all subsets are processed, one iteration is completed.

The utilization of the OSEM method allows significant acceleration of the image reconstruction process. In case of using 16 subsets, for example, the images convergence process would be accelerated by a factor of 16 as compared with MLEM, considerably reducing the computation time required for the reconstruction^(8,16)^.

Due to the great computational effort required by the iterative methods during image reconstruction, as they require the repetition of projection and back projection mathematical operations, images correction and updating, such methods were not considered as a convenient solution for clinical use in the past, as they required several minutes to generate a single image. However, due to computer evolving over the past years, the iterative reconstruction methods became clinically useful, demonstrating to be a powerful option over conventional methods of filtered back projection. Additionally, such methods have been improved with the development of efficient models and algorithms with increasingly faster reconstruction.

The images produced by the iterative methods are also different from those generated by analytical methods, particularly on what concerns the amount of noise, target-tobackground ratio, resolution and detailing of the image. Besides the immense gain in convergence time, the iterative algorithms present the advantage of allowing the easy incorporation of physical process models that influence total quantification, such as photon attenuation in the tissue and scattering, as, for example, blurring compensation as a function of the distance between the patient and the detector and reduction of lines generated by the star effect in the areas with high photon counts^(7,12,14,16-18)^.

The value of the iterative methods depends upon each situation and the needs from each department, so it is better for some tasks than for others. However, there is not a consensus on the parameters to be utilized, such as the most appropriate number of iterations or number of subsets. In spite of its several advantages over the analytical methods, the OSEM method generally used to be the main choice of specialists, and not the *ordinary users'* involved in the clinical routine. Currently, many apparatuses have got this type of method as a standard feature^(7)^.

Some authors advocate that the choice of parameters during images reconstruction using iterative methods does not depend on the physical characteristics of the patients. However, after the patient is injected with the radiopharmaceutical, the gamma radiation (photons) encounter a natural barrier (the patient) that absorbs many of them. Such attenuation depends a lot on the individual's body dimensions. This is so true that, according to Arrighi^(19)^, one tends to increase the value of injected activity for heavier patients. Therefore, the greater the patient's body mass, the greater the attenuation of photons by the tissues will be, and greater will be the detectors' difficulty to capture the photons. This also causes changes in the way by which the images should be later processed.

Thus, considering that radiation attenuation by tissues varies according to the physical characteristics of the patient, and also considering the WHO classification with respect to BMI, the present study aimed at evaluating the BMI influence in the selection of parameters of the iterative OSEM algorithm to be utilized in myocardial perfusion imaging reconstruction. For such a purpose, MPI studies performed in a public hospital were analyzed and processed by using the iterative OSEM method with different combinations of number of iterations and number of subsets, aiming at determining a combination of parameters of the iterative method for the different BMI classes, according to evaluation by nuclear cardiology specialists with respect to the most appropriate clinical quality of diagnosis.

## MATERIALS AND METHODS

The study was developed in accordance with all ethical principles governing the utilization of confidential data from patients. For that reason, throughout the development of the present study, no data related to the patients was disclosed. The present study was submitted to the Committee for Ethics in Research of the institution, being duly approved under reference No. 151/11 (104-DEFI/132-CES).

The collected data were those recorded by the medical team: patients' name, date of birth, weight and height, the last two data utilized to calculate each patient's BMI.

Data collection was carried out in three phases. The first phase involved 43 patients submitted to MPI between Jan 1st, 2011 and March 2nd, 2011. The second phase involved 40 patients who underwent MPI in the institution in the period between Jan 15th, 2011 and Jan 31st, 2011, as well as on April 27th, 2011. In the third phase, three patients who underwent MPI on Jun 4th, 2011 and Jun 5th, 2015, were selected. Thus, the initial study sample comprised 86 patients, 23 of them in the normal BMI class (B), 32 in the overweight class (C), 20 with level I obesity (DI), and 11 patients with level II obesity (DII).

The target population in the present investigation comprised adult patients from both genders distributed in BMI classes B, C, DI and DII (Table 1) who underwent MPISPECT with both phases - stress and rest -, utilizing the "one-day protocol" at a public general hospital.

**Table 1:** Classification according to BMI.

Classification		BMI (kg/m^2^)
Underweight (A)	Thinness class III	< 16.00
	Thinness class II	16.00-16.99
	Thinness class I	17.00-18.49
Normal (B)	Eutrophia	18.50-22.99
		23.00-24.99
Overweight (C)	Pre-obesity	25.00-27.49
		27.50-29.99
Obesity (D)	Obesity class I	30.00-32.49
		32.50-34.99
	Obesity class II	35.00-37.49
		38.00-39.99
	Obesity class III	≥ 40.00

The BMI classes A (underweight) and DIII (level III obesity) were not considered in the present study as there were no available patients of such classes. Also, neither the clinical indications for the exams, nor the respective scintigraphic findings were taken into consideration for the purposes of the present investigation.

During the analysis and selection of patients, the following exclusion criteria were considered: pediatric patients; patients whose exams did not present the acquisitions in stress or rest; patients whose images presented motion artifacts, overlapping of anatomical structures in relation to the heart, low target-background ratio; patients whose images could not be appropriately imported into the research workstation; and patients whose image acquisition protocols were different from those considered as "standard" in the institution.

The scans were performed using a Siemens E-Cam DSR dual head apparatus with two detectors and LEHR (low energy high-resolution) collimators with parallel holes. The radiopharmaceutical utilized was ^99m^Tc-sestamibi with activities of 10 mCi in the rest phase, and 30 mCi in the stress phase. The radiopharmaceutical injection was made by means of puncture into the antecubital vein of the patients. For the scan, all patients were positioned in dorsal decubitus, with the arms extended over the head, and the imaging was performed with electrocardiography synchronization by means of the forward backward by thirds method, in auto-tracking mode, with 8 frames and 10-cycle acceptance window. The SPECT acquisition mode was step-and-shoot, with noncircular orbit, with a total of 64 projections (32 for each detector). The time for each projection was approximately 25 seconds, leading to a total duration of 30 minutes. The pixel size was 6.59 mm and zoom was 1.45.

The data collected in the investigated institution were recorded on CDs and imported into a Hermes Medical Solutions^TM^ Compact Server workstation for analysis of the images and image processing. Dell 1907FP monitors were utilized for images visualization.

Considering the need to keep the anonymity of the patients, for each one of them a unique identification code was created without disclosing their respective data. Each one of the codes took into account the date of collection (phase in which the data were collected - A, B or C); BMI class (B, C, DI or DII); and the sequence in which the exam was collected (by numerical order).

Considering that the iterative methods allow for several, different combinations of number of iterations and number of subsets, it was initially established 16 arrangements, formed by the combination of 2, 4, 6 and 8 iterations with 2, 4, 8 and 16 subsets. From that point, three patients were randomly selected for images processing with each one of the possible options, with one patient being from class B, one from class C and the third one, from class DII.

The resulting images were internally analyzed by a restricted team of three nuclear medicine specialists who selected the six arrangements that presented the best image quality. Such a selection was based on a global analysis of the images quality, essentially considering technical characteristics, target-background ratio, as well as changes in the images (deformations), which generated loss of information. Thus, the most promising sets of reconstruction parameters could be defined.

**Figure 1:**
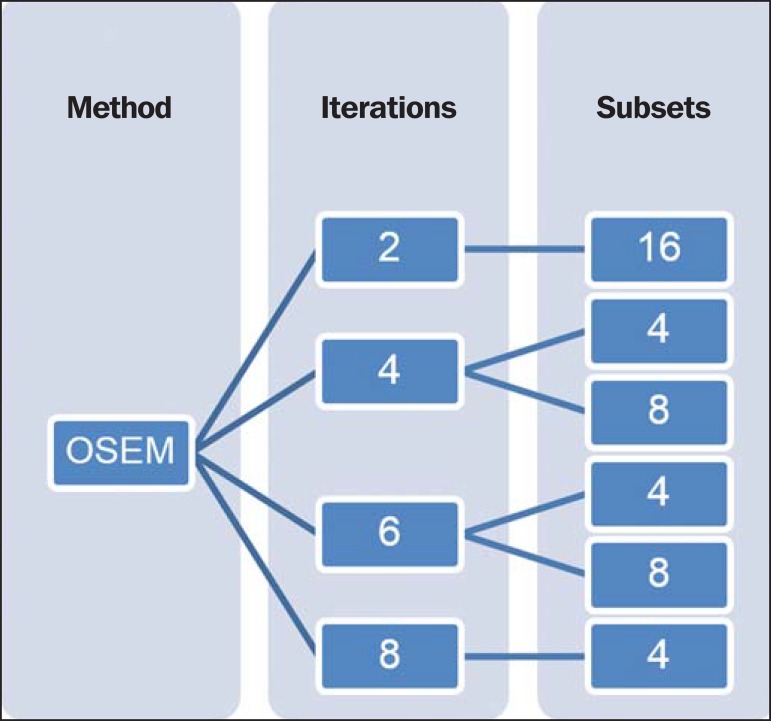
Defined combinations of iterations and subsets numbers for the study.

Once the OSEM parameters were defined, as shown on Figure 1, and the exclusion criteria were applied, 28 individuals from the initial sampling were selected, with 7 individuals from each one of the BMI classes. The minimum age was 37 years and the maximum age was 80 years (mean 59 years). Table 2 presents data regarding patients' gender, age and BMI.

**Table 2:** Patients' gender, age and BMI.

Class	Gender		Numberof patients	Age				BMI			
	Male	Female		Minimum	Maximum	Mean	Median	Minimum	Maximum	Mean	Median
B	5	2	7	37	68	59	61	18.82	23.66	21.84	22.84
C	7	0	7	48	77	62	59	25.16	29.94	27.25	27.10
DI	3	4	7	53	72	59	58	30.49	34.11	32.33	31.89
DII	3	4	7	49	80	58	54	35.06	39.16	37.02	36.79
Total	18	10	28	37	80	60	59	18.82	39.16	29.61	30.21

For each patient, six different processing runs were performed, one for each selected iteration/subset combination. In total, 168 reconstructions were performed. In the images reconstruction, the data were tomographically processed in the orthogonal, transverse, sagittal and coronal planes, with the Quick Cardiac 1.3.1^TM^ tool available at the workstation, and visualized in print mode (option QPS 3, splash mode) with 4× zooming and without the cardiac contour automatically generated by the software. Attenuation correction options were not utilized.

The generated images were saved in portable network graphics (PNG) format, which has the advantage of not presenting with high level of losses when the images are manipulated. Each file was named with the code of the respective patient and the iteration/subset combination utilized in the reconstruction. A new worksheet was also created for organization of the images, considering that the workstation allows for filing each image in a separate folder in the hard disk^(20)^.

With the objective of removing all data that could identify the patients, the images resulting from the processing were manipulated with the Microsoft Paint^TM^ software. The images' characteristics such as resolution, brightness and contrast were not changed.

**Figure 2:**
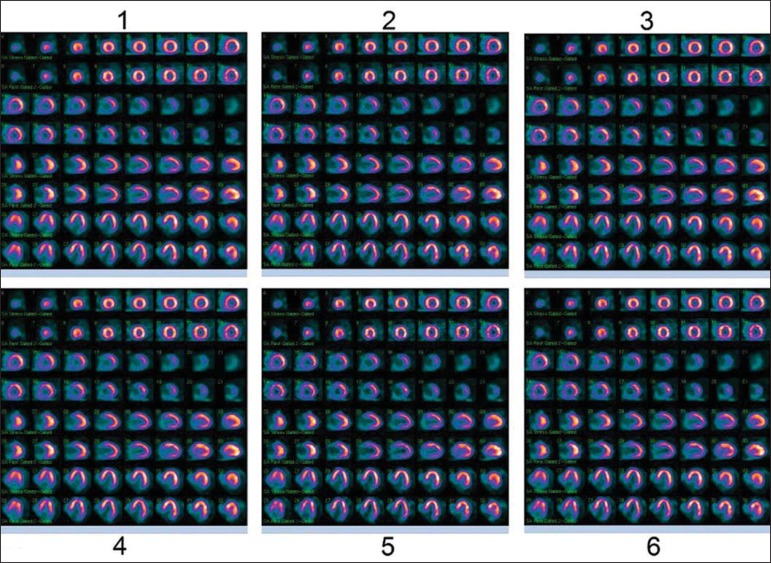
Display of reconstructed images on the A3 size sheet.

For each patient, the image sets resulting from the six processing runs were saved as a single PNG file, and printed on photographic quality A3 format paper, in landscape mode, as shown on Figure 2. Each set was numerically identified from one to six according to the position on the sheet. The images were randomly positioned, thus avoiding that the physicians, upon analyzing the images, could be influenced to always select the same iteration/subset combination.

For the medical evaluation of the images, an instrument was developed, where the participating physicians indicated the number corresponding to the three image sets which they considered as having the best quality in terms of diagnosis. The images were analyzed by ten nuclear medicine specialists, five of them Portuguese physicians from different nuclear medicine departments of the cities of Coimbra and Porto, and five Brazilian physicians from the cities of Florianópolis and Porto Alegre.

A scoring was applied to the combinations selected as the best by the specialists. For the first option from each observer, the score was five points; for the second option, three points; and for the third option, the score was one point. The maximum scoring that an arrangement could reach was 315 points.

## RESULTS

**Figure 3:**
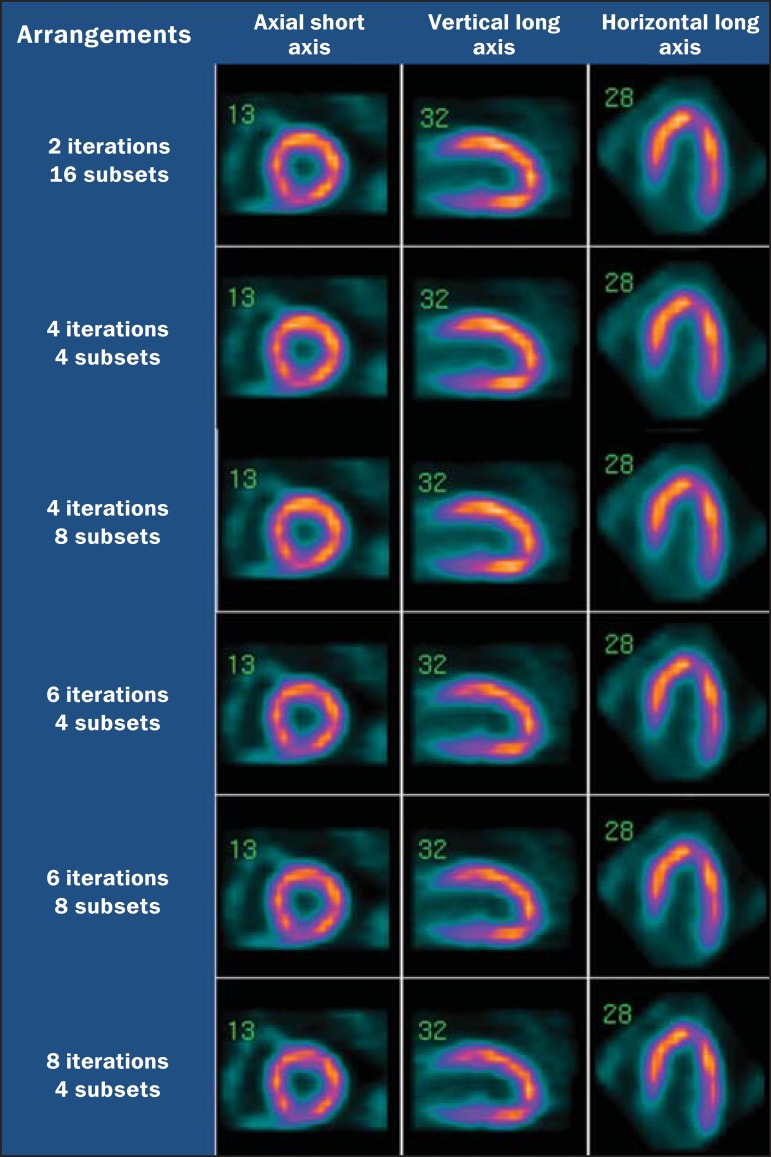
Axial short axis, vertical long axis and horizontal long axis resulted from image processing.

**Figure 4:**
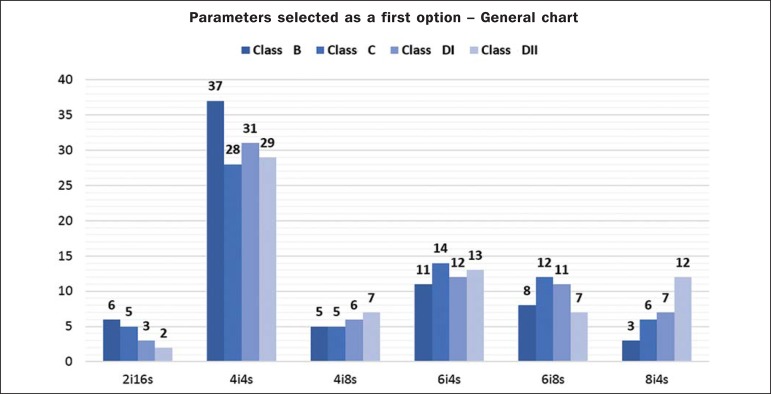
General chart showing the parameters selected as a first option by the specialists.

Figure 3 presents a comparison of the images in the three usual planes resulting from processing utilizing the six defined arrangements of iterations and subsets. Figure 4 shows the number of times each parameter was selected as being that which produced the best images, distributed among the BMI classes.

In general, the arrangement of four iterations with four subsets was the most frequently selected as that producing the best images. Considering that each arrangement could be selected up to 280 times, such combination represents 44.64% of the options, more than twice the number of times that the combination of six iterations with four subsets, the second most frequently selected combination, were chosen. None of the remaining combinations outstood from the others, and the least frequently selected combination was 2 iterations with 16 subsets (5.71%).

Considering the analyzed BMI classes, the preeminence of the combination of four iterations with four subsets in relation to the others is more expressive in class B, representing 52.86% of the choices. In classes C, DI and DII, the combination of four iterations with four subsets represents, on average, 42% of the choices. The preeminence in relation to the other combinations remained in all evaluated classes, as shown on Table 3.

**Table 3:** Overall parameters scoring.

Class	Scoring - evaluations					
	2i16s	4i4s	4i8s	6i4s	6i8s	8i4s
B	78	219	90	129	53	61
C	66	183	90	148	81	62
DI	67	184	73	160	63	83
DII	48	197	57	163	52	113

**Figure 5:**
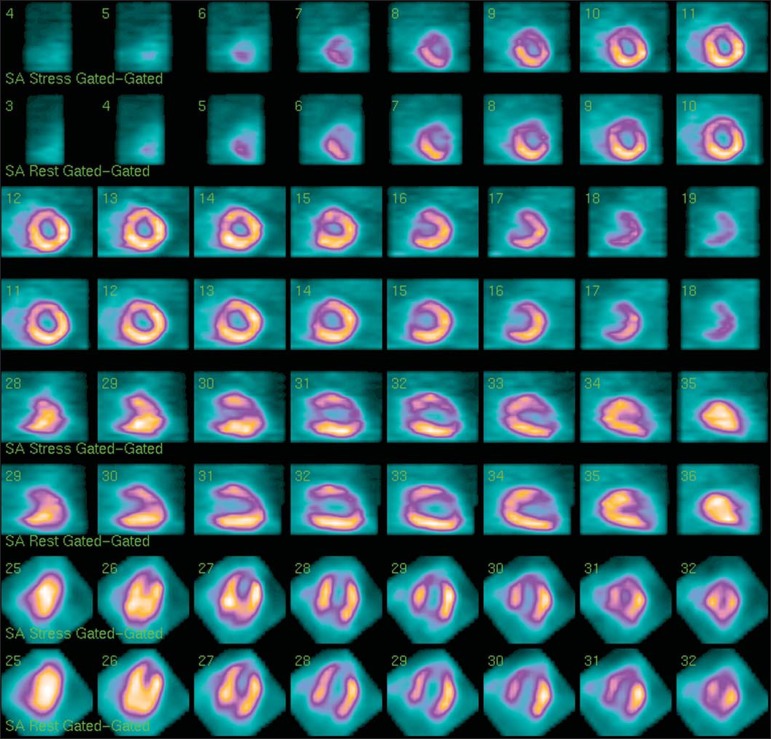
Resulting images from processing using four iterations and four subsets.

As the scoring was applied to the specialists' choices, the combination of four iterations with four subsets was still preeminent, and such combination generated the best images. Figure 5 shows the result from the image processing with that combination of parameters. In addition, as the scoring is applied, one realizes that the combination of six iterations with four subsets also becomes expressive. However, for class DII, the arrangement of eight iterations and four subsets had a high score as compared with the other combinations.

## DISCUSSION

Based on the analysis of the arrangements scoring, it is possible to observe that, for higher BMI classes, image-processing utilizing the OSEM method and a higher number of iterations outstands in relation to the others. Additionally, it is possible to observe that, in spite of the variations in the number of iterations of the selected parameters combinations (four and six), the number of subsets of the preeminent combinations remains constant as four.

The increased BMI implies increase amount of body tissues, leading to higher attenuation of the radiation emitted by the patient. On the basis of the present study results, one understands that for patients in lower BMI classes, as they are less radiation attenuating, the arrangement of four iterations with four subsets seems to be sufficient for the images processing. generating quality results. As the BMI increases, the more the result will undergo changes related to tissue attenuation, possibly leading to a higher number of iterations necessary for image convergence.

According to Slomka et al.^(21)^, with the increase in the number of image updates (product of the number of iterations and subsets), the spatial resolution increases, but with the occurrence of noise. Thus, it is important to optimize parameters in order to obtain the best image quality. According to those authors, two to four projections are normally used for each subset (generating 16 to 32 subsets, respectively, considering a 64-projection acquisition).

According to Seret^(22)^, it is interesting that less experienced users always utilize eight subsets and, minimally, two iterations for MIP reconstruction. However the author warns that such a low number of iterations result in fainter images, with low noise level, but with a poor contrast resolution, particularly in poorly perfused areas. In addition, according to the author, the subsets should have, at least, four projections. A lower number of projections would lead the OSEM algorithm to a divergent tendency, not being able to reach an optimal solution. Therefore, 64 projections can be divided into a maximum of 16 subsets.

Thus, according to the results observed in the present study, the increase in the number of iterations performed in the images processing assures a images reconstruction in terms of diagnostic quality.

During the investigation, the time required for each combination to complete the image processing was not considered to be relevant and was not even measured, as it was always short. However, no significant differences were observed, which might imply disadvantages in the utilization of the method in the clinical context.

It is also interesting to observe that no attenuation and scattering correction methods were utilized, as this could significantly influence the images quality after reconstruction. The adoption of such correction methods might change the results and suggest that other arrangements result in better images.

It is also known that every institution utilizes protocols adapted to their particular realities, possibly with variations in each service routines. Such differences, such as the color scale utilized in the images processing, for example, lead to variations in the selection of the most appropriate parameters.

Although the SPECT technique is based in a simple form of image acquisition, the whole image reconstruction process is quite complex. In MPI, as well as in other types of studies, several factors influence the final results.

Thus, such factors give more agility to the nuclear medicine service and, in addition, reduce errors associated with interoperator variabilities, as the processing procedures (as well as those to perform the scans) are similar. The utilization of an appropriate and controlled routine allows for the reduction of the doses delivered to the patient, as well as to the professionals acting at the service, since the number of repetitions of scans is reduced with the utilization of optimized practices.

Thus, the service achieves adequate diagnostic quality, as the interpretation of images does not depend only of the clinical knowledge of the reporting specialist, but mostly on the whole exam process, from the preparation of the patient, the scan itself and the data manipulation for the images reconstruction.

## CONCLUSION

In a general analysis of the scoring generated by the medical evaluations, the present study allows the conclusion that the use of the iterative OSEM method set to perform four iterations in four subsets seems to generate images with the best diagnostic quality in cases of patients with normal BMI, overweight and obesity levels I and II, followed by the combination of six iterations and four subsets in the higher obesity classes.

The utilization of optimized parameters seems to play an important role in providing for images reconstruction with better diagnostic quality, thus ensuring the diagnosis and consequential appropriate and effective treatment for the patient.
